# Changing interventions in farm animal health and welfare: A governmentality approach to the case of lameness☆

**DOI:** 10.1016/j.jrurstud.2022.12.004

**Published:** 2023-01

**Authors:** Lewis Holloway, Niamh Mahon, Beth Clark, Amy Proctor

**Affiliations:** a School of Environmental Sciences, University of Hull, Cottingham Road, Hull, HU6 7RX, UK; b Centre for Rural Economy, School of Natural and Environmental Sciences, Newcastle University, Newcastle upon Tyne, NE1 7RU, UK

**Keywords:** Farming, Cows and sheep, Lameness, Governmentality, Health and welfare, UK

## Abstract

Lameness is a significant health and welfare issue in farmed animals. This paper uses a governmentality approach, which focuses on how a problem is made governable, to examine an emerging ‘ecology of devices’ introduced to intervene in, and attempt to reduce, on-farm incidence of lameness. These devices are associated with advisers who work with farmers on-farm; they enact lameness as a governable entity, are tools to assess the existence of lameness against established norms, and prescribe actions to be taken in response to evidence of lameness. In doing this they subjectify farmers and advisers into seeing and responding to lameness in particular ways. Using concepts of governmentality alongside other perspectives on the power relations and the simplifications and complexities involved in interventions in animal health and farm practice, the paper draws on in-depth research with advisers including vets and other paraprofessionals who work with farmers, and their cows and sheep. It explores how this set of devices introduces particular techniques and practices in lameness management, and produces farmer and adviser subjectivities. It then explores some of the problematics of this mode of governing lameness, including analysis of the limitations and unintended consequences of attempts to simplify lameness management. The paper concludes by arguing that its approach is valuable in analysing ongoing intensification of interventions in farming practices and in understanding the limits of such interventions and the unanticipated divergences from expected conduct.

## 1 Introduction

There has been considerable social scientific interest in the health and welfare of farmed animals. This has examined, amongst other things, the problematics of biosecurity (e.g. Enticott, 2008; Hinchliffe et al., 2016), efforts to address endemic disease (e.g. Bellet et al., 2021; Shortall and Brown, 2021; Wynands et al., 2021), and debates around animal welfare (e.g. Buller and Morris, 2003; Buller and Roe, 2018; Weary and Robbins, 2019). Attention has been paid to the roles of different actors, including farmers and vets (e.g. Enticott, 2012; Naylor et al., 2018; Shortall et al., 2018; Holloway et al., 2022; Mahon et al., 2021), and to relevant policy contexts (e.g. Shortall and Calo, 2021). In this paper we develop a novel perspective by investigating an emergent governmental mode of intervention in endemic conditions in animals on UK farms, focusing specifically on lameness in cows and sheep as a phenomenon with significant welfare implications (e.g. McLennan, 2018) and reductions in animals’ and farms’ productivity (e.g. Nieuwhof and Bishop, 2005).

The paper focuses on farm advisers (including vets, hoof trimmers and other paraprofessionals) in relation to lameness because of their key roles in encouraging farmers to make changes and in implementing on-farm treatment strategies Early social scientific work focused on them as facilitators of ‘innovation diffusion’ and ‘technology transfer’, perspectives now regarded as simplistic (e.g. Padel, 2001; Ruttan, 1996). More recently, the roles of advisers have been theorised in more complex ways, looking at the importance of relationships between advisers and farmers, and between advisers and other advisers, in guiding and regulating farm management (e.g. Proctor et al., 2012; Phillipson et al., 2016), how advisers’ professionalism and expertise are produced through social learning and communities of practice (e.g. Landini et al., 2017; Nettle et al., 2018; Phillipson et al., 2016), and how advisers’ increasing use of protocols in areas such as disease management is tempered by the demands of complex farm environments. Enticott (2012) describes this as a ‘local universality’ based on ‘situated expertise’ explaining how protocols are made to work in varying situations.

Specifically in relation to the management of endemic health conditions, the roles of advisers in mediating specialist knowledges and influencing on-farm practices have been studied, including discussion of how adviser and farmer knowledge-practices interact in specific situations of uncertainty to produce new practices (see e.g. Enticott et al., 2011; Woods, 2013b; Woods, 2013a; Clarke and Knights, 2018; Shortall et al., 2016; Woodward et al., 2019; Enticott, 2012; Enticott and Franklin, 2009). Advisers’ changing roles as part of an emergent government of lameness has been noted. Key here is an argument that vets should move away from ‘fire-fighting’ emergency problems, to administering preventative, whole population health planning (Kaler and Green, 2013; Clarke and Knights, 2018; Woods, 2011). This can be understood as part of a wider concern with biosecurity and its attempts to anticipate and address the risk of future incidences of threats to animal health (Bingham et al., 2008; Donaldson, 2008; Hinchliffe et al., 2016).

This shift is one we align with a particular kind of agricultural governmentality, an approach drawing on Foucault’s analyses of power (Miller and Rose, 2008). This approach sees lameness enacted asa measurable phenomenon, determines an expertise which is brought to bear on lameness, and guides the conduct and subjectivity of farmers and others so that they become responsibilised in assessing, recording and intervening in individual- and population-level incidences of lameness. We focus, then, on how lameness is framed as a governable problem, and is made governable through what we refer to as an ecology of devices: a set of inter-related tools intended to drive particular kinds of intervention. Singleton and Law describe devices as purposeful contrivances involving ‘strategies that work more or less repetitively to order, sort, define and arrange a heterogeneous but relatively discrete social and material field’ (2013: 260). The devices we discuss in this paper are thus involved in the constitution of a field of lameness management and work to affect the interventions made in this field. The paper addresses the following three questions. First, we ask how emerging ways of addressing lameness can be conceptualised by drawing on governmentality, and how governmental relations function alongside other forms of power discussed by Foucault. Second, focusing on advisers’ perspectives and on different devices, we ask how the subjectivities of advisers and farmers are constituted in relation to the mode of governmentality and the ecology of devices we describe. Third, we ask how attempts to make lameness the subject of this kind of governmentality to simplify its management can produce unexpected problems or ‘divergent conduct’ (Bear and Holloway, 2019). The paper thus uses a governmentality approach to provide insights into the roles of advisers and an emerging ecology of lameness-management devices, and to explore the value and limitations of emerging preventative modes of intervention.

Next, we briefly discuss the issue of lameness and outline how it has been approached as a problem for farming. We then discuss governmentality, situated alongside other forms of power discussed by Foucault. We also introduce ideas about complexity as a means of framing our discussion of how farmers are being guided towards particular ways of intervening in lameness, and how such attempts work in practice. After outlining our empirical approach which involved detailed interviews with advisers, the paper explores two areas. First, we focus on perspectives on the devices which have been introduced to guide farmers’ conduct and subjectivity in relation to lameness. Second, we discuss advisers’ comments on implementing this regimeon farms in the north of England, focusing on how these attempts to intervene become problematic in the context of individual farms and farming systems, with unanticipated and perverse outcomes. We conclude by arguing that a governmentality approach to animal health and welfare conditions such as lameness is valuable in evaluating ongoing changes in how such conditions are intervened in, how such interventions are bound up with specific technologies, practices and the production of subjectivity, and how in practice interventions can be limited or have unintended consequences.

## 2 The problem of lameness

Lameness, ‘any variation/defect which causes abnormalities in an [animal’s] gait and can include a variety of leg and foot conditions’ (Cutress, 2020: unpaginated), is a persistent welfare and productivity problem (e.g. Fraser and Broom, 1997; Webster, 1995; Kaler and Green, 2009; Tunstall et al., 2019). It is regarded as a ‘production condition’, associated with particular ways of farminganimals, and despite being neglected (Rioja-Lang et al., 2020) is important because it can have effects on animals’ productivity (e.g. growth rate, fertility or milk yield) (see Bennett and Ijpelaar, 2005). As Leach et al. (2010) argue, farmers are motivated by a concern for animal welfare, and take pride in having healthy animals. Yet lameness is regarded as a particularly complex condition to deal with. It has multiple causes (including infection and injury, and those related to genetic inheritance and bodily conformation), is associated with complex relationships between animals and farming environments/systems, and because it can be recorded and addressed in different ways (see, for example, Wynands et al. (2021) regarding US dairy farming, Holzhauer and van Egmond (2021) regarding Netherlands dairy farming, K. Best et al. (2020a, b) regarding Australian sheep farming and Terrell et al. (2017) regarding US and Canadian beef cattle). Further, it is argued that lameness can become normalised, and thus pass unnoticed, on some farms, a situation referred to by Croyle et al. (2019: cited in Wynands et al., 2021) as ‘barn blindness’.

As a result, social scientific studies of lameness argue that farmers face significant barriers in putting lameness treatment and prevention measures into practice (C. Best et al., 2020a; Horseman et al., 2014; Wynands et al., 2021). Attention is thus paid to how to make interventions more effective. Main et al. (2012) and Whay et al. (2012), emphasise the importance of advisers in generating effective change. Farmers keeping cattle and sheep are increasingly encouraged to use a set of devices – including scoring systems, action plans and monitoring technologies – to reduce lameness. Farmers are thus enrolled by advisers into new sets of practices, and changing relationships with advisers and other institutions, which produce interventions in the lives of farmed animals with the aim of reducing, if not eliminating, lameness. Lameness is thus produced in particular kinds of farming systems and environments, and by the framing of lameness through veterinary and farming knowledge-practices and an ecology of devices which renders it amenable to, and stimulate, certain interventions. We next consider a conceptual framing for understanding the production of lameness as a governable entity.

## 3 Governmentality, simplification and complexity

The concept of governmentality has underpinned several analyses of health and disease in farmed animals and plants (e.g. Higgins and Dibden, 2011; Enticott et al., 2021; Curran, 2001). We draw on conceptualisations of governmentality, or the ‘conduct of conduct’ (Miller, 2008; Miller and Rose, 2008; Li, 2007b; Murdoch, 2006; Lemke, 2002), to discuss how lameness has become framed as a governable problem requiring the input of specific kinds of expertise, measurement and calculation, the creation of devices, and programmes of action. This framing attempts to both raise the profile of lameness as a problem for farmers and other actors so that they become enrolled into addressing it in particular ways, and to simplify how it is addressed by providing tools for assessing lameness in individual animals and populations of animals, along with instructions for what to do in response. We also draw on concepts of complexity and simplification to explore how attempts to simplify lameness management produce new kinds of complexity (Mol and Law, 2002). We use this to show how a government of lameness does not always have its intended effects.

Grounded in a Foucauldian understanding of how power relations produce phenomena in society (e.g. Foucault, 2007; Burchell et al.,1991), as well as science and technology studies (e.g. Latour, 1999), governmentality approaches describe how calculative techniques enact phenomena, rendering them amenable to interventions, and shaping action to drive ‘improvement’ in often quite mundane aspects of life (Miller, 2008; Srinivasan, 2014; Li, 2007b). An understanding of phenomena as enacted is important to this perspective (Latour, 1999) as phenomena are co-constituted with devices which produce them through measurement, and are intended to drive particular interventions, aligned with a programme of ‘improving’ the collective qualities of a population. A governmentality perspective considers why conduct needs conducting in a situation, how a phenomenon is problematized, how forms of expertise are produced and implemented, how problems are made amenable to intervention, and how tools facilitating intervention are deployed (Miller and Rose, 2008).

This approach to governmentality can be seen in relation to other Foucauldian perspectives on how power functions productively in a society, including notions of sovereign and disciplinary power, bio-power/biopolitics, and pastoral power. We simplify greatly here in outlining these perspectives on power. While sovereign power relates to the power held by the state over the bodies of subjects, disciplinary power involves the establishment of institutions which encourage the internalisation of norms by subjects, leading to desired behaviours and practices. Clinics and schools, for example, produce healthy, educated subjects with internalised norms relating to how they should behave (Schirato et al., 2012). Biopower, and biopolitical relations, relate to strategies which focus on the ‘life’ of individuals and populations. Described by Nealon (2007) as an ‘intensification’ of disciplinary relations, it describes the fostering of the phenomena of life, such as birth, death and morbidity rates, in order to optimise the productivity of a population (Foucault, 1990). Rose (2007: 53) describes how biopower represents a combination of measuring and recording techniques which produce knowledge about people, and strategies for using that knowledge to effect change at population level; it is ‘a multitude of attempts to manage their life, to turn their individual and collective lives into information and knowledge, and to intervene on them’. Biopower involves normalisation: quantifiable norms mediate between individuals and populations, and depend on systems of measuring the population characteristics and assessing the extent to which individuals adhere to or deviate from them (Nealon, 2007). Interventions then aim to produce greater adherence to norms. While Foucault’s accounts of biopower are anthropocentric, its focus on life processes arguably makes it a useful tool to deploy in studies of nonhuman life as well (e.g. Holloway et al., 2009; Wolfe, 2012). In this paper, for example, the cows and sheep are viewed alongside humans as subject to these kinds of intervention. Finally here, the idea of pastoral power was used by Foucault (e.g. 1982) to describe how some individuals take on roles of responsibility and care towards other individuals and groups, and exercise that responsibility through seeking ‘confession’ from individuals which produces knowledge about themselves which can be used in directing them towards a ‘better’ life (see e.g. Cole, 2011; Pandian, 2008). As part of technologies of power, then, some people thus become responsible for the lives of others, and for shaping their subjectivities and practices in specific ways, through pastoral relationships. Pastoral power can be seen as part of biopolitics: it works at individual and population levels, focusing on care for individuals while involving the direction of a wider population (Pandian, 2008).

These interconnected perspectives provide ways of describing what is happening within the ecology of devices we explore. Although sometimes seen in terms of an historical series of transitions between modes of power, it is more accurate to describe how different kinds of power relation emerge, working alongside and within other kinds, so that in different examples multiple modes of power relation can be observed. The government of lameness can thus be seen as drawing together aspects of the different modes of power outlined above. Although we focus on governmentality, other modes of power are referred to as they intersect with governmental relationships. Governmentality, as the making of something governable, involves both a discursive problematisation which establishes a phenomenon as something to be appraised and intervened in, and an assemblage of practices, tools and technologies which enact that appraisal and intervention (Miller, 2008; Miller and Rose, 2008; Briassoulis, 2019; Li, 2007a, 2007b). It is associated with an emerging faith in quantification as the way to evaluate the world, and, importantly, with a co-emergent trust in new forms of what Miller and Rose (2008) refer to as mundane expertise, i.e. expertise relating to everyday practices that nevertheless require the deployment of specialist skills and knowledges. Miller and Rose (2008) provide a useful summary of governmentality. It involves first, the production of particular kinds of truth; second, the presence of authorities which can speak that truth and urge intervention on its basis; and third, processes constituting the subjectivity of people who become expected to change their thinking and practices in accordance with this truth. In the case of lameness, we argue that a particular ‘truth’ concerning the condition is produced through the introduction of devices which measure and record it, that authorities including vets become invested with an expertise in identifying lameness and driving change based on new ways of constructing it as a phenomenon to be intervened in, and that farmers become subjectified into particular ways of ‘noticing’ and addressing lameness in their animals.

It is important to note, however, that a governmental regime is not ‘closed’ and that programmes of intervention might have unanticipated consequences. Li (2007b) identifies several points. First, the human and nonhuman objects of government are not passive; they are ‘actants, dynamic forces in social life, constantly surprising those who would harness them’ (p.277). In relation to farmed animals, their ‘liveliness’ is a factor in how government happens and in the success or otherwise of interventions (see Barua, 2016; Collard and Dempsey, 2013). At the same time, people implicated in a regime of governmentality, for example as attempts are made to enrol them into a particular mode of intervention, may not, or may not be able to, comply (see also Foucault, 2007). For Foucault, attempts to govern always produce resistances and unintended consequences (see e.g. Nealon, 2007; Holloway and Morris, 2012), and attempts to intervene in the parameters of life through government and biopolitics may not always be effective as ‘life’ inevitably escapes their techniques (Foucault, 1990). As Foucault put it, ‘[if] one says to a population “do this”, there is not only no guarantee that it will do it, but there is quite simply no guarantee that it can do it’ (Foucault, 2007: 71). People may be recalcitrant in the face of attempts to govern their conduct. Second, the techniques, knowledges and devices available for intervention are always partial and incomplete, and thus are imperfect in implementing interventions. Third, the ‘anti-political’ tendency of governmentality (see Barry, 2002; Higgins and Dibden, 2011) can effectively preclude the asking of wider ‘political’ questions about situations defined in terms of technical expertise and intervention. Li concludes that situated studies are needed of what occurs when attempts at government happen, and on the intended and unintended effects of a governmental regime.

This suggests that programmes of government which aim to simplify phenomena by framing them in particular ways and articulating a mode of intervention, are in practice subject to the complexities associated with the actual situations in which interventions are attempted. As Mol and Law (2002) argue, simplification is not simply reductive, but also productive, for example of ways of knowing something, of subjectivities, and of new complexities. As such, simplification and complexity are co-produced (Mol and Law, 2002; Latour, 1999). Although simplification within governmentality can be presented as a rational attempt to reduce, measure and intervene in the complexity of something, the unknown and unpredictable qualities of that something mean these actions are unlikely to be complete. Attempts to simplify how phenomena are understood, and thus amenable to particular kinds of intervention, can produce new and unexpected complexities.

Studies of farming situations have thus examined what happens when interventions intended to simplify and conduct on-farm practices have been introduced, and which have met with divergencies resulting from the meeting of different, incompatible knowledge-practices and the messiness of actual farm environments, and unexpected outcomes (Bear and Holloway, 2019). These studies include examples which examine tensions between scientific attempts to frame and intervene in farming practice and farmers’ own situated knowledges of their farms (Tsouvalis et al., 2000; Holloway and Morris, 2008; Holloway, 2005; Visser et al., 2021). Others have focused on the complexities of deploying veterinary knowledges in farming (see Hinchliffe et al., 2016): e.g., Shortall and Brown (2021) and Shortall and Calo (2021) focus on the negotiations involved in enrolling farmers into a disease eradication, Maye and Chan(2020) examine the unevenness of farmers’ responses to implementing biosecurity protocols, and Merrill et al. (2019) look at farmers’ willingness and ability to comply with biosecurity measures. Similarly, Moya et al. (2021) and Enticott (2012) look at how veterinary and on-farm expertise is negotiated in relation to animal health measures, in the situated complexity of specific farms.

The discussion of governmentality and other modes of power relation, and of complexity and simplification, provide us with a set of tools for examining farm advisers’ perspectives on emerging ways of monitoring and intervening in lameness. They are valuable in thinking about how devices structure responses to lameness and are associated with the production of adviser and farmer subjectivity and expertise relating to lameness. They also help in considering both what might be more effective interventions, and how such interventions might be limited in practice by the messiness of on-farm situations. Against this background we turn now to discuss our case study of lameness.

## 4 Research methods

We draw on interviews with advisers with a specialist interest in lameness, including vets, consultants and paraprofessionals such as hoof trimmers. They were part of a larger research project focusing on different endemic diseases in UK cattle and sheep farming (see Holloway et al., 2022), involving research with farmers in the North of England who had one or more of dairy, beef or sheep enterprises, and the advisers who work with them, between September 2019 and March 2021. Research was affected by Covid-19 restrictions imposed in the UK from March 2020 (Holloway, 2020): all except two adviser interviews were conducted remotely. In this paper we utilise a set of detailed, in-depth interviews with nine advisers who had a specialist interest in lameness (Table 1). We recruited vets from large veterinary practices specialising in cattle and sheep work, along with those working for smaller practices which had been mentioned in farmer interviews. Other advisers were searched for using professional databases or had been mentioned by farmer interviewees. We focus on these advisers as they commented in depth on the devices associated with lameness, were themselves enrolled into the government of lameness, and were involved in enrolling farmers too. Although this is a small sample, first, interviewees represented a close-knit community of such advisers who (although there will necessarily be differences in perspective and approach) broadly share a professional milieu and work within a similar ecology of devices, and second, the research focused on exploring the issues with the advisers in depth instead of attempting to expand the breadth of coverage. This approach reflects discussion of appropriate sample sizes in qualitative research with groups with a shared background, which can provide meaningful and in-depth insights into their discursive frameworks and practices (see e.g., Boddy, 2016; Vasileiou et al., 2018). Interviews focused on interviewees’ professional roles in relation to endemic health conditions, their relationships with farmers, the history of their interventions in endemic health conditions, and the problems and limitations of those interventions. Similarities between interviewees’ comments, and their reference to similar set of devices and interventions, provided confidence that an accurate picture of an emergent government of lameness was drawn. Interviews were recorded and transcribed, and then coded with Nvivo software, using a codebook developed to assist analysis of this dataset.

We first discuss the ecology of devices which has emerged as a way of framing and reducing lameness. In doing this we show how lameness has been made governable, and also indicate how other forms of power relation are part of this, describing how particular subjectivities are produced through the government of lameness. Second, we explore the problematics of this framing of and intervention in lameness, showing how in practice there are important limitations and unintended consequences.

## 5 An ecology of devices: framing and governing lameness

Advisers identified an ecology of devices promoted as ways of reducing lameness in cows and sheep. To explore key aspects of the power relations involved in the government of lameness, we draw particularly from an interview with a veterinary consultant (A14) who discussed the Healthy Feet Programme (HFP), a device created by the UK’s farmer-funded Agriculture and Horticulture Development Board (AHDB), and which is designed for use with dairy cows (ahdb.org.uk/healthy-feet [accessed April 5, 2022]). We describe the HFP, connecting it to a wider ecology of inter-related devices, and show how this enacts lameness as a governable entity (Miller and Rose, 2008). We go on to explore how farmer and adviser subjectivities are produced, and how different kinds of power are evident in the government of lameness.

### 5.1 The Healthy Feet Programme and an ecology of devices

The HFP involves farmers working with mentors (e.g. vets), using a risk assessment tool to drive changes to on-farm practices to reduce the incidence of lameness, and using a Hoof Care Field Guide to, according to the AHDB’s *Introduction to the Healthy Feet Programme*, ‘recognise, treat and record lesions properly’, to correctly mobility-score cows (see below), to use correct footbathing, and to ‘cost out herd lameness and calculate a cost-benefit for changes considered’ (2018: unpaginated). The HFP involves ‘participatory epidemiology’: farmers and advisers create lameness data and make a farm ‘lameness map’, develop an action plan and monitor its implementation, benchmarking against other farms and regularly reviewing data and targets. As noted by Miller and Rose (2008: 67), requiring someone to collect data is part of subjectification; ‘[m]aking people write things down and count them … is itself a form of government of them, an incitement of individuals to construe their lives according to such norms’ . Here this takes place in relation to the government of lameness in cows, with the enrolment of farmers in participatory epidemiology starting to construct their subjectivity, in terms of what they are expected to know and do vis-à-vis lameness. The HFP, reflecting accounts of the functioning of governmentality in specific fields (Miller and Rose, 2008; Li, 2007b) illustrates a government of lameness which focuses on framing lameness in a particular way, establishes appropriate modes of intervention, and involves the kind of normalisation process described by Nealon (2007) whereby farmers are encouraged to compare their performance with others. The HFP is thus a device which has agency, affecting the subjectivities and practices of both farmers and advisers, and driving specific kinds of intervention in the care of cows. Associated with an institution (the AHDB) established to promote ‘progress’ in farming, and which thus plays an important role in the creation of particular kinds of farmer subject, the HFP is also an outcome of a biopolitics which seeks to measure and intervene in an aspect of bovine life, leading to ‘improved’, more productive bodies in ways similar to those described in other agricultural and non-agricultural fields (e.g., Srinivasan, 2014; Holloway, 2005). The HFP is similar to other programmes, such as the ‘Five Point Plan’ (5 PP) aimed at sheep farmers (see C.Best et al., 2020a), which govern lameness through imposing structured routes towards reduction. Farmers are encouraged to undertake five actions, including culling persistently lame sheep, quarantining sheep with infectious lameness, using effective treatments, vaccinating, and preventative management of sheep and their environment. As with the HFP, the 5 PP influences farmer subjectivity, along with on-farm practices, by encouraging farmers to recognise lameness as a problem of the ‘life’ of their sheep, and to intervene in response. Foucault’ s concepts of disciplinary and biopower (e.g., Foucault, 1990; Foucault, 2007), are here applied to animal (instead of human) bodies, and together are key to the government of lameness. These modes of power are evident in the existence of institutions like the AHDB and in interventions focusing on the health and productive performance of living bodies.

Interventions driven by devices such as the HFP and 5 PP are themselves dependent on an ecology of supplementary devices, technologies and techniques, such as mobility scoring processes, which provide a dataset which enacts lameness and makes it visible so that specific interventions can be prescribed (Miller, 2008). These devices guide the conduct of farmers and advisers in specific ways and are crucial in making lameness governable. The government of lameness is thus something increasingly dependent on data collection devices and the centres of calculation they correspond with, with these viewed as producing objectively better lameness management. Singleton and Law’s (2013) discussion of the functioning of devices in a field, noted above, is evident in the following description of the various tools contributing here to the government of lameness.

AHDB mobility scoring for cows, for example (ahdb.org.uk/knowledge-library/mobility-scoring-how-to-score-your-cows [accessed April 5, 2022]), requires scorers to rate each cow on a scale from 0 (good mobility) to 3 (severely impaired mobility). A mobility scoresheet is provided with descriptions and photographs for each score, and farmers create data by recording scores (either themselves or employing an independent mobility scorer) for each cow, each month. Particular attention is paid to cows scoring 2 and 3, but each score is associated with recommended interventions: e.g., for cows scoring 0, ongoing preventative foot trimming is recommended, for those scoring 3, urgent attention and nursing is required. Adding a further layer to this mode of government, mobility scorers are themselves regulated and subjectified, e.g. by enrolment in the Register of Mobility Scorers (RoMS) (roms.org.uk [accessed April 5, 2022]), an ‘independent self-regulatory body which encourages the widespread use of standardised, independent mobility scoring conducted by trained and accredited scorers … to a set of professional standards’. Registration through professional bodies and institutions such as RoMS acknowledges and certifies the mundane expertise (Miller and Rose, 2008) of mobility scorers, making them key actors in the government of lameness through creating a subjectivity as qualified professionals authorised to play a specific role.

The HFP and mobility scoring together draw attention to the importance of practices and techniques of hoof trimming to interventions in lameness. Further government of this mode of intervention is evident; we use dairy cows as an example again. First, the process of trimming is standardised, with a series of defined steps driving action. The ‘Dutch five step’ trimming method (ahdb.org.uk/knowledge-library/trimming-cows-feet-the-five-step-dutch-method [accessed April 5, 2022]) is an important example. The first three steps are defined as ‘functional’ and are used for all cows; steps 4 and 5 are ‘therapeutic’, used to address specific lameness problems. Second, as noted, the creation and use of data is key to governmentality, and this is exemplified in the way ‘today’s modern hoof trimmer’ is encouraged to record and share data through devices such as the All4feet software (www.all4feet.uk [accessed April 5, 2022]). All4feet allows trimmers to upload records, recommendations and images while on-farm, which can be shared with other actors, including farmers and vets. Farmers can access a dashboard of records pertaining to their cows and be prompted to take specific actions in response to trimmers’ recommendations. Third and finally, in a similar way to RoMS members, trimmers themselves can become enrolled into and subjectified by a government of foot trimming which involves certifying and authenticating their expertise. For example, the Cattle Hoof Care Standards Board (CHCSB) was established in 2016 to set standards for trimming, ‘against which hoof trimmers can be robustly audited’ through unannounced on-farm checks: ‘a field-based audit also allows assessment of how workplace challenges are recognised and addressed, which are important aspects of professionalism’ (www.hoofcarestandards.co.uk [accessed April 5, 2022]). The CHCSB produces the embodied, hard and dirty work of hoof trimming as an arena of professionalism, according with descriptions of how governmentality creates new kinds of mundane expertise associated with data production and standardised practices (Miller and Rose, 2008). Together with the other devices mentioned here, the CHCSB, acting as a disciplinary institution, produces the professional subjectivity of hoof trimmers by requiring them to keep records. This drives the adoption of specific practices in relation to footcare and lameness by both trimmers and farmers, as both are subjectified by these devices into becoming responsive to formal data which enacts lameness as a quantified entity.

### 5.2 Interventions and subjectivity in the government of lameness

Having outlined these devices and articulated them in terms of a government of lameness, we turn to how advisers discussed them to illustrate how lameness is enacted as a governable entity, associated with particular subjectivities and interventions (Miller and Rose, 2008). We draw in particular froman interview (A14) with a consultant, and focus on the HFP. The discussion directs attention to how the HFP governs lameness, both framing it in particular ways and directing specific interventions, through a process of dialogue between vet and farmer which depends on the production of particular subjective positions, and produces knowledges about, and actions regarding, lameness. Building on Foucault’s concepts of disciplinary, pastoral and biopower relations, this government of lameness focuses on improving nonhuman bodies, via processes of human subjectification and intervention.

The consultant discussed the HFP in terms of a relationship between simplification and complexity, saying that:

‘[i]mproving the knowledge isn’t the thing really. It’s all about simplifying it. And that’s what the Healthy Feet Programme is there to do […] it is all about trying to simplify what is a complicated set of processes’.

The HFP was described as having two elements – the:

‘ … four success factors and the lameness map. And it’s very visual … the four success factors, I’ll tell you what they are just so you get the framework of it. It’s to have low infection pressure. It’s to have a robust foot. It’s to have low forces on the feet and that’s cow comfort and cow flow. And it’s early detection, prompt and effective treatment … And you can write this on a piece of paper’.

This piece of paper forms the basis of the lameness ‘map’, a new device in its own right. These tools (the four success factors and the map) are then utilised by the consultant in a reflexive mode referred to by some interviewees as a ‘coaching’ approach, in order to help farmers articulate the issues on their farm. The consultant described a model conversation between themselves and a farmer, showing how they drew the issues out from the farmer’s knowledge of their farm and cows:

‘You ask them, “what do you think might be involved in infection pressure?“, and they’ll say, “Oh, well, footbathing”, “yeah, yeah, well done … what’s involved in having a robust foot?“, “well I guess nutrition comes into that”, “Yeah, yeah it does”, “and breeding”, “yeah, yeah. You’re right, spot on. What comes into low forces?“, “Well, I guess the cows lying down because of cubicle comfort”, so they get that’.

Through this kind of questioning dialogue, the consultant adopts a pastoral subjectivity (Foucault, 1983) which enrols and subjectifies the farmer into a framing of lameness which then renders this problem of nonhuman bodies amenable to the interventions proposed by the HFP. The mundane expertise of the interviewee is deployed as part of the government of lameness, through their pastoral relationship with the farmer, which coincides with the disciplinary, institutionalised governmentality of the HFP and thus the AHDB, and with the biopolitics of focusing on dimensions of bovine life and performance.

The consultant described the HFP as a ‘balanced scorecard’ approach, an approach used in business management (Kaplan and Norton, 2005). It was described as ‘a bit like doing a SWOT analysis … so you’ve got your four corners. And you look at your strengths and weaknesses in each of those corners and it helps you know where you need to be working.’ In using this kind of device, the farmer becomes subjectified in relation to the approach as they work on themselves along with their cows) (Schirato et al., 2012). The consultant discussed how a farmer will have:

‘ … used that balanced scorecard methodology to simplify lameness. So, you’ve got infection pressure. You’ve got robust foot. You’ve got forces, which is split into lying times and cow flow. And then you’ve got early detection, prompt effective treatment, which is how quickly are you finding your lame cows and what have you got in place for treating them and are you treating them correctly? And then your balanced scorecard is you look at the four main disease types and they are digital dermatitis, white line disease, and sole ulcers. And sole bruising as well, yeah? I mean, it’s all on pictures. So you establish this with farmer, you know, “what do you see with your cows’ feet?” And you show them the pictures. He’s like, “Oh I see a lot of that. I see a lot of that” … and then this balanced scorecard business is, right, okay, we get quite a lot of digital dermatitis, but we don’t get much white line disease. But we get quite a lot of sole ulcers … if you’ve got accurate records you can use the records, but often it is just, what do you feel? It’s a participatory epidemiology approach, which is basically just asking the farmer what they think. And then you draw your diagram, your map. So on this particular farm, this is where you want to concentrate … this is where you’re going to get your best benefits. So it simplifies what is a very complicated thing’.

The HFP, and the ecology of associated agencies and devices including mobility scoring, RoMS, CHCSB and All4feet, is doing a number of different things here, contributing to an overall government of lameness through the coincidence of different modes of power relation, including subjectification through disciplinary institutions and devices, the biopolitics of specific ways of measuring and intervening in the life processes of individual animals and (herd) populations, and the pastoral techniques of ‘coaching’ farmers. Our example thus draws together and adds to descriptions of disciplinary, pastoral and biopower (Rose, 2007; Schirato et al., 2012), framing these as part of a wider government of lameness (Li, 2007b; Miller and Rose, 2008) which deploys interventions in human subjectivity to enact lameness as a governable phenomenon and drive specific interventions. As such, programmes like the HFP conduct the conduct (Lemke, 2002) of advisers and farmers in relation to lameness. The HFP frames lameness according to a simplifying schema, which is intended to provoke reflection and to drive interventions. An authoritative ‘truth’ about a complex problem like lameness as an entity which can be simplified and mapped according to four ‘success factors’ and devices like the balanced scorecard, is established, and farmers are subjectified into viewing their cows, farms and practices through a dialogue in which they are encouraged to frame lameness in a particular way. In doing this, the farmer is subjectified in relation to the programme, as they are positioned as needing to have the complexity of lameness simplified, as they are ‘coached’ through pastoral dialogue with a consultant and enrolled through participatory epidemiology, are encouraged to record and use data, and have their interventions determined by the programme. The HFP drives the collection of data and the production of inscriptions – including balanced scorecards and lameness maps – which act to carry something of the lameness experienced corporeally by cows into a framework for intervention, and into disciplinary processes of benchmarking and normalisation across farms. And, finally, the HFP, like Pasteur’s extension of the laboratory onto the farm (Latour, 1983: cited in Murdoch, 2006), extends a government of lameness over space from institutions such as the AHDB and veterinary practices, into farms.

## 6 Governing lameness: complexity and divergence

In the previous section, we outlined an ecology of devices associated with the government of lameness, linking together the production of mundane expertise, programmes for intervention, and a set of devices which enact lameness. We focused on the coincidence of different kinds of power relation in the government of lameness, on how lameness was rendered as a governable entity linked with specific techniques and practices of measurement and intervention, and on the production of farmer and adviser subjectivities. Next, we discuss some of the problematics of attempts to govern lameness in this way, reflecting our earlier discussion of the limitations of governmental and other modes of power (Foucault, 1990, 2007; Burchell et al., 1991; Li, 2007b) and of the relationships between simplification and complexity (Mol and Law, 2002). We again draw on the perspectives of farm advisers in considering how the government of lameness is practised, and the new complexities and divergence from intended consequences that can result. We introduce four issues: first, the relationship between attempts to simplify lameness government and the complexities of farm environments, lively bodies and farmer responses to devices such as the HFP; second, the need for vets to continue to mould farmer subjectivity and practice so that they ‘see’ lameness and assess and respond to it ‘correctly’; third, the unintended consequences of interventions in lameness; and fourth, that this mode of governing lameness produces (and facilitates) further disciplinary monitoring of farmers’ and other actors’ subjectivities and practices.

### 6.1 Simplifying devices and complexity

Lameness devices intended to simplify farm practices can generate further rounds of complexity, following Mol and Law (2002). They are in tension with the complexities of actual farm situations and the liveliness of the animals involved. As one vet said, ‘it can be quite difficult, especially with animal health and welfare stuff, to try and impose the same thing on all our farms because they are all very, very different’ (A4). A consultant, making reference to the 5 PP, explored how the attempt to implement this device produces new complexities in practice.

‘For lameness, the five-point plan is a really nice framework of what people can do. But in reality, how you implement that shifts depending on what information you’ve got. So what is the incidence and prevalence of disease? What’s the weather doing? Where can you move [sheep] to? … So, all of that, you can imagine it like a supercomputer, you’re trying to take in all of these different factors and influences using some form of framework and trying to make the best … or as I generally refer to it, the least-worst decision […] And also, the reality, which is these group of ewe lambs were away from the farm, and so they weren’t very easy to catch. I mean, it’s lovely to say, “oh, yeah, you’ve got to catch them when one limps”. It’s like, yeah, go on try in a massive field with no handling system. So, it’s recognising that best practice is one thing … it’s just layering on the knowledge with actually what is practical on the farm’ (A17)

This excerpt emphasises, mirroring Enticott’s (2012) discussion of local universality, how the complexities of specific farms and animal bodies and populations as ‘lively commodities’ (Barua, 2016; Collard and Dempsey, 2013), alongside environmental conditions, make implementing something like the 5 PP complex, demanding responsiveness to specific, changing material circumstances and available information. The embodied, lively capacities of sheep, and specific farming conditions (a large field lacking handling facilities) combine to disrupt the intended intervention.

In addition, the requirements of devices can be off-putting for some farmers in spite of claims that they simplify lameness management through standardisation and protocols. While arguing that the HFP, for example, is an effective intervention, advisers acknowledge that following it is demanding. In response, the AHDB created a simplified ‘HFP-lite’. An adviser explained that ‘we know [the HFP] works. But if we say to a farmer “you have to tie in for a year and this is what we’re going to do”, they might just think “but I don’t want all of that. I don’t want to commit”. Whereas the Healthy Feet Lite is meant to be a simple one-off intervention and then a follow-up 12 months later’ (A11). In this example, reflecting Mol and Law’s (2002) comments on the implications of simplification, the idea that the HFP is a way of simplifying lameness management is rendered problematic by how, for some farmers, it still involves a degree of complexity and commitment they are not prepared for so that they resist the subjectification it imposes. As such, in a dialectical relationship between complexity and simplification, a still simpler version of the HFP is created as a way of enrolling more farmers into this government of lameness.

### 6.2 Making lameness noticeable

According to some advisers, some farmers still need encouragement to notice lameness where it has become normalised as part of farming. Governing lameness implies working on farmer subjectivity in order to re-orient perspectives around different norms and practices regarding seeing and intervening in the condition. Lameness needs to be thus enacted as something governable (Miller, 2008). This is supplemented here by the adoption in some cases of a pastoral approach (Pandian, 2008): a vet described their ‘gently, gently approach … you see all the animals and say, “do you think that cow is a bit sore on her feet? Shall we have a chat about that?”, I think that softly, softly approach is quite important … I do think it’s a barrier because they’re also frightened … of losing their contract’ (A11). This mention of anxieties about contracts introduces another aspect of the apparatus of lameness government. Dairy farmers’ contracts to supply milk buyers are dependent on welfare assurance programmes which set maximum acceptable levels of lameness in herds, and which can be seen as another part of the administration of welfare and of the subjectification of farmers into a way of knowing about and intervening in their animals’ lives (see e.g. Escobar and Demeritt, 2017). An interviewee who conducted welfare assurance inspections for a national organisation discussed how they used inspections to co-produce mundane expertise about lameness and mobility scoring with farmers, again adopting a pastoral role in learning with farmers about the situation on specific farms. As they explained, ‘in the dairy scheme we’re looking at mobility, so we have to see animals moving … and we do the scores together, so I’m going “I’m scoring this as a 2, because of this, this and this”, so we talk about what we’re trying to score’ (A19). The same interviewee, however, also talked about the assurance scheme in terms suggesting that it was also an expression of disciplinary power, so that, for example, ‘ … they have to have mobility scoring four times a year by preferably a RoMS scorer who’s got training. There’s prescriptive things in terms of what they should be scoring and how they’re dealing with that, so have scores of 2 or 3 they must put in some plan or have been treated … I’ll be making sure the medication records backs the fact that they’ve been treated and then they have a plan going forwards’ (A19). Here, attention is drawn, within lameness government, to the importance of RoMS certification and to the prescription of certain actions which must be taken, and be recorded as taken, by farmers in the event of lameness scoring ‘revealing’ problems.

However, another interviewee said that ‘[The HFP is] a way to get farmers into taking lameness more seriously, and also being more aware of it, because I think that one of the things that a lot of lameness experts would agree on is … it has been so prevalent that you don’t realise how much lower level lameness you have … If you have a lame cow that is scoring a 2 or a 3, you are so used to seeing 2s and 3s that it’s harder to pick up on a 1 that is going to become a 2 and a 3 maybe in a couple of weeks’ time’ (A5). Here, reference is made to an unintended effect of mobility scoring, whereby instead of animals being regarded as lame and needing treatment, first, their lameness becomes normalised and is not ‘seen’, and second, those animals scoring 1, which are showing early signs of lameness, are not identified as problematic at all. The HFP is, then, a way of countering the effects of a scoring system that produces its own problems (counter-intuitively, it can make lameness *less* visible), and is dependent on scoring being calibrated ‘correctly’ by actors performing it. In relation to this latter point, the problematics of subjective assessment being used to produce quantified scores which then become represented as objective records of lameness prevalence were highlighted by some advisers, emphasising that part of their role was to guide farmers to use the scoring system correctly. One vet described their work as aiming to produce ‘robust data’, which can be used to motivate dairy farmers to take appropriate action, but that this depends on sensitising farmers to the correct calibration of their scoring. As they said,

‘so you’ve got 0 which is perfect mobility, 1 which is imperfect, 2 is lame, 3 is very lame. Now, everybody pretty much agrees on the 3s … but it’s the threshold between 1 and 2. Now, a lot of people, if they’re waiting for it to be definitely lame, they’re a couple of weeks late, so we need to be picking them up really early. So what I find is actually my early score 2s are a lot of people’s 1s, but actually they’re the animals you need to pick up. So I often get called harsh. I call it realistic.’ (A11)

Mobility scoring as a device, then, is subjective and potentially unstable but does have significant effects as an intervention in the subjectification and practices of farmers. It is deployed to drive further interventions as data accumulates recording the percentages of animals with different scores, which can lead to preventative or therapeutic actions and/or to the maintenance or loss of welfare assured status (and thus to the continuation or loss of a contract with a buyer). In this example, again, attempts to simplify the government of lameness using devices such as the HFP leads to unexpected new complexities (Mol and Law, 2002), in this case associated with an inability to fully control farmers’ perceptions and practices.

### 6.3 Lameness devices and perverse outcomes

Welfare assurance schemes and mobility scoring are further associated with examples of divergence from the intended practices, subjectivities and outcomes of lameness government. In these cases, the schemes and the devices they deploy are shown to themselves become actors in the situations they are involved in (Singleton and Law, 2013); they are not neutral creators of records of an on-farm situation but active mediators in those situations in unanticipated ways. One vet recorded how, ‘I think there obviously has been this drive by the milk buyers. They have been putting in that they want to have X amount of lameness or less … I think that’s actually been quite a negative thing because if you’re told that you have to have less than 20% lameness, you’ll just say you’ve got less than 20% lameness … I have farmers who would be way above 20%, but when they submit their scores they’re 20%’ (A11). This adds to understandings of the unanticipated consequences of governmental regimes and their attempts to simplify conduct (Li, 2007b; Mol and Law, 2002) in illustrating how efforts to simplify can lead to new complexities associated with the deployment of specific devices which make particular demands on the responses of people expected to be compliant with them. In examples like this, farmers have become subjectified into thinking about lameness in the intended way, as part of a disciplinary schema which sets norms around the acceptable prevalence of lameness, yet they simultaneously subvert this discipline by submitting data which in the vet’s view inaccurately represents the actual prevalence of lameness, knowing that to submit an accurate record would lead to sanctions.

Similarly, a consultant working with sheep farmers described how farmers would respond to questions about levels of lameness; ‘one of the first questions I asked them … was “how many lame sheep have you got?“, they said, “Well, under the 5%“. They had learnt [that if they said more than] 5% that then there was a reaction’ (A13). Here again the subjectification and discipline of the farmer in terms of what they have learned that they need to say has the unintended effect of them only confessing to misleadingly low lameness prevalence. In this example, the consultant performed their own mobility scoring which produced a figure of almost 10% lameness, despite the farmer’s use of antibiotics to try to address this issue. In these cases, where quantitative mobility scoring is dependent on subjective assessments, the assurance scheme itself has a perverse effect on what is ‘seen’ and recorded on farm to ensure that the conditions of the assurance scheme are met. A consultant added to this problematisation of lameness scoring, saying that ‘it needs to be flexible enough rather than saying “Well we want all lameness in sheep flocks to be 2% or less”. And? Or if you go into a flock and say, “your lameness is 10% at the moment. We’re going to pay you if you can get that down to 5%“. So, the farmer can go down to 5% but he might just do that by throwing a bucketload of antibiotics at them and doing blanket treatments’ (A18). In these cases, quite perverse outcomes can be identified, where the attempt to use devices such as lameness scoring as part of a normalisation process produces unexpected negative outcomes in terms of the fabrication of data to meet required standards, or practices such as the over-use of antibiotics which, while superficially addressing a lameness problem, contribute to rising concerns about antimicrobial resistance (see e.g. Morris et al., 2016).

### 6.4 Lameness and divergent conduct

Finally, governing lameness produces a need to further monitor farmers’ activities, at the same time as farmers are made responsible for monitoring their animals through the collection and use of data. The All4feet programme was mentioned above as a device used by foot trimmers to share information. Such devices can also be used to check whether farmers are using the data appropriately. As one foot trimmer explained,

‘ … as soon as I’ve been to a farm, I download their report … and they can log on to their farm details, so that they can actually see what I have done and what the problems are […] The beauty of the programme is that I can actually see when the farmer logged on, so I know if they are looking at the reports or if they’re not. And like I say, I’ve got some that are really, really good and switched on and look at it all the time. And I’ve got plenty that don’t bother’ (A3)

All4feet is thus involved in the surveillance and subjectification of farmers, and identifies ‘good’ farmers who conform, and those who resist through a lack of engagement. It can monitor if they are using data as they should be, and extends the ability of advisers to act on farming practices. The comment shows the limits of this kind of government as identified by Li (2007b), acknowledging that some farmers do not access the available information. Adding to this sense that the government of lameness can be limited in practice, one vet described how devices which record what is being done on farms can also identify where other actors in the government of lameness, in this case foot trimmers themselves, are not compliant with guidelines on how trimming should be done. The vet discussed how it was important for them to be able to access the trimmers’ records because ‘[y]ou can monitor whether they’re following the protocols … That’s a challenge I have on one of the farms at the moment. The trimmer isn’t following the protocols. The trimming is the main cause of lameness we’re seeing at the moment’ (A11). In examples like this, the device allows identification of divergence from what is expected, at the same time as providing tools for driving compliant practices and creating ‘good’ lameness subjectivities.

In sum, while interviewees in our research described how the government of lameness can be successful in reducing lameness, attempts to intervene in lameness in this way are also associated with effects that limit its effectiveness (Li, 2007b; Nealon, 2007). These include tensions between attempts to simplify lameness management and the complexities of farm situations and farmer responses, the sometimes perverse effects of implementing certain devices, and the need to continually make noticing of lameness happen and to monitor and intervene in different actors’ practices.

## 7 Conclusions

This paper has developed a novel approach to thinking about how lameness in cattle and sheep is being addressed, inspired by work on governmentality and other modes of power described by Foucault (e.g., Miller and Rose, 2008; Li, 2007b), and on descriptions of the nature of devices used to constitute and order specific fields of activity (Singleton and Law, 2013) to describe how an emerging ecology of devices is involved in the constitution of farmer and adviser subjectivities and the implementation of new kinds of on-farm practice. It thus contributes to and extends previous work addressing adviser-led interventions in situations related to animal health and welfare issues on farms (e.g., Enticott, 2012; Moya et al., 2021). Drawing on detailed empirical research with farmer advisers in the north of England, we showed how in relation to particular aspects of the government of lameness, governmentality as an expression of power relations is entangled with disciplinary power, biopower/biopolitics and pastoral power. Our use of governmentality as an approach has been extended by thinking about the simplification-complexity relationships and problematics associated with efforts to guide conduct (Mol and Law, 2002) as a way of conceptualising some of the limits to attempts to govern lameness in the ways we described.

The government of lameness involves an ecology of inter-related devices including systems for assessing lameness, quantitative and qualitative representations of lameness which enter into centres of calculation concerning the condition, monitoring and recording tools, normalisation schema, and frameworks which attempt to drive particular practices. Describing this ecology illustrates how lameness becomes framed and problematized as a governable entity, requiring both certain kinds of intervention and the production of subjectivities and mundane expertise. The paper showed how the deployment of particular devices and normalisation techniques involved attempts to work on the subjectivities of farmers (and advisers), with the intention of internalising particular ways of ‘seeing’ lameness and, as a result, making specific interventions driven by detailed protocols. This accords with arguments that adviser roles are expected to change, as they are enrolled into discourses of preventative intervention and whole farm health planning (Woods, 2011). Advisers are increasingly and reflexively adopting roles they describe in terms of ‘coaching’, and which can be described as pastoral, in that they involve soliciting responses from farmers encouraged to reflect on their own situations and practices, with the intention of ‘improving’ the health and welfare of their animals.

In our investigation a key question has been to consider why lameness persists as a significant problem for cattle and sheep in UK farming, when interventions exist which are seen by key actors as potentially highly effective in reducing the prevalence and severity of the condition. The paper has shown how an emerging government of lameness cannot be completely ‘closed’, particularly in light of the unexpected complexities linked to attempts to simplify conduct in relation to lameness. As Mol and Law (2002) have argued, simplification is productive, leading to new kinds of knowledge and practice, as well as to new complexities. As we show here, it also produces divergent and/or perverse conduct. Complexities associated with the liveliness (Barua, 2016; Collard and Dempsey, 2013) of the animals involved, and with (ironically) the attempts to simplify and standardise lameness management, mean that implementing this government of lameness is thus not straightforward. As a result, there are divergences from expected conduct and practice along with unexpected and sometimes perverse outcomes, including resistance to aspects of the subjectification driven by the various interactions within this ecology of devices. This analysis contributes to explaining how lameness remains an ongoing problem, not simply resolved by the implementation of new kinds of intervention.

Our work provides a perspective on the role of advisers and farmers in interventions in farmed animal health and welfare which suggests a need to pay further attention to two areas in further studies of such topics. First, the conceptual framework of governmentality is useful as a way of thinking about how a condition such as lameness, which has been seen in different ways at different times, is here enacted as an entity which can be measured and intervened in specific ways, associated with the production of particular kinds of knowledge, subjectivity and practice (Miller, 2008; Miller and Rose, 2008; Li, 2007b). This sense that such conditions are constituted differently, at different times and different places, and in relation to different knowledge and power relations, is important in further studies of the roles of advisers as they are both part of the making and promulgation of particular disease constitutions, and are themselves subject to wider disciplinary and subjectification processes (e.g. within veterinary science, in policy and in the regulation and assurance of animal health and welfare). This might also exemplify an ‘anti-political’ tendency (Barry, 2002) to ask bigger questions about the conditions in which farmed animals are kept and which might be regarded as ‘lameness-ogenic’ (Holloway et al., 2022). Alongside this, there is a need to further explore advisers’ relationships with farmers, in the ongoing processes of making diseases governable entities and intervening in ways driven by particular instances of government and through different ecologies of devices.

Relatedly, the sense that the government of lameness (and other endemic conditions) is not, and cannot be, ‘closed’ (Li, 2007b), warrants ongoing attention. The further intensification of attempts to govern on-farm animal health and welfare issues suggests a prevailing view that ongoing rounds of the kinds of intervention we discuss in this paper will be effective. For example, the approach to lameness government (along with other endemic health conditions) described in this paper is likely to become further established in England through its embedding within the Department for Environment, Food and Rural Affairs’ (Defra’s) Animal Health and Welfare Pathway, with interventions in lameness being recognised in potential future government financial support for farming (https://www.gov.uk/government/publications/animal-health-and-welfare-pathway [accessed November 8, 2022]). Drawing on the findings of this paper, it is important to continue to investigate the implications of such interventions for changing and recombining modes of disciplinary power, biopower and pastoral power within the government of animal health and disease, to trace their effects on farmer and adviser subjectivities, knowledges and practices, to examine the effectiveness of these interventions in their own terms, and to explore the new complexities and problems they produce despite and because of interventions which intend to reduce endemic disease.

## Figures and Tables

**Table 1:** Adviser interviewee roles.

Interviewee no.	Gender	Role
A3	male	Cattle hoof trimmer
A4	female	Veterinary consultant
A5	female	Levy board staff member
A11	female	Vet
A13	male	Farm consultant
A14	male	Veterinary consultant
A17	female	Farm consultant
A18	female	Farm consultant
A19	female	Assurance scheme assessor

## Data Availability

Anonymised data will be shared via the UK Data Archive at the completion of the project which has funded this research.
